# Development and Validation of a Diagnostic Model to Predict the Risk of Ischemic Liver Injury After Stanford A Aortic Dissection Surgery

**DOI:** 10.3389/fcvm.2021.701537

**Published:** 2021-09-23

**Authors:** Maomao Liu, Wen Tan, Wen Yuan, Tengke Wang, Xuran Lu, Nan Liu

**Affiliations:** Center for Cardiac Intensive, Beijing Anzhen Hospital, Capital Medical University, Beijing, China

**Keywords:** ischemic liver injury, Stanford A aortic dissection, cardiac surgery, risk prediction model, intensive care unit

## Abstract

**Objective:** To define the risk factors of ischemic liver injury (ILI) following Stanford A aortic dissection surgery and to propose a diagnostic model for individual risk prediction.

**Methods:** We reviewed the clinical parameters of ILI patients who underwent cardiac surgery from Beijing Anzhen Hospital, Capital Medical University between January 1, 2015 and October 30, 2020. The data was analyzed by the use of univariable and multivariable logistic regression analysis. A risk prediction model was established and validated, which showed a favorable discriminating ability and might contribute to clinical decision-making for ILI after Stanford A aortic dissection (AAD) surgery. The discriminative ability and calibration of the diagnostic model to predict ILI were tested using C statistics, calibration plots, and clinical usefulness.

**Results:** In total, 1,343 patients who underwent AAD surgery were included in the study. After univariable and multivariable logistic regression analysis, the following variables were incorporated in the prediction of ILI: pre-operative serum creatinine, pre-operative RBC count <3.31 T/L, aortic cross-clamp time >140 min, intraoperative lactic acid level, the transfusion of WRBC, atrial fibrillation within post-operative 24 h. The risk model was validated by internal sets. The model showed a robust discrimination, with an area under the receiver operating characteristic (ROC) curve of 0.718. The calibration plots for the probability of perioperative ischemic liver injury showed coherence between the predictive probability and the actual probability (Hosmer-Lemeshow test, *P* = 0.637). In the validation cohort, the nomogram still revealed good discrimination (C statistic = 0.727) and good calibration (Hosmer-Lemeshow test, *P* = 0.872). The 10-fold cross-validation of the nomogram showed that the average misdiagnosis rate was 9.95% and the lowest misdiagnosis rate was 9.81%.

**Conclusion:** Our risk model can be used to predict the probability of ILI after AAD surgery and have the potential to assist clinicians in making treatment recommendations.

## Introduction

Ischemic liver injury (ILI) is a clinical syndrome characterized by acute and dramatic increases in serum aminotransferase to a level of more than 10 times the upper limit of normal, which is caused by insufficient oxygen and blood delivery to the hepatocytes (1–3). The underlying etiologies leading to ILI are cardiac, circulatory or respiratory failure (2–6). The incidence of ILI ranges between 1 and 12% in intensive care unit (ICU) (3, 6–9), and may be even higher in patients with cardiogenic shock (3, 6, 10). The all-cause mortality rate is 25 ~ 73% (1, 2, 5–7), of which more than 50% occurred during ICU stay (2, 3, 7, 11). The surgical procedures for Stanford A aortic dissection (AAD) are complicated, and the situation is changeable during the operation. Despite improvement in perioperative management and surgical techniques, the malperfusion syndromes (i.e., ischemic liver injury) are often present as sequelae of general ischemia (12, 13).

Reliable prognostication in ILI after AAD surgery provides clinicians with helpful information about diagnosis and short-term and long-term outcomes. ILI is the most common cause of dramatic elevation of serum transaminase levels in ICU (5–7, 14, 15). Clinicians should try to recognize the incidence of ILI as early as possible to avoid complications of the hepatic injury which may trigger progression of multiorgan failure (5–7, 16–18). However, Denis et al. found that, in cardiac intensive care unit, the diagnosis of ischemic liver injury may be delayed under some circumstances (18). A delay in diagnosis may signify delayed treatment and worse outcomes. In addition, the occurrence of ILI has significantly increased in-hospital mortality of those critically ill patients (7, 11). By far, the main clinical management of ILI is the cure of the underlying diseases in the ICU (3, 4), it rarely has specific treatments to improve the liver function (7, 11). Statin treatment may be protective against the development of ischemic liver injury (1, 10, 19), but the therapeutic potential after occurrence of ILI and an overall survival benefit was not identified. Therefore, it is important to predict whether ILI occurs after AAD surgery.

Although several risk factors (20–23) have been identified are associated with the occurrence of post-operative ILI, to our knowledge, barely any study has illustrated these factors in patients with AAD surgery. Therefore, accurate prediction in patients with ILI after AAD surgery remains a challenge. The aim of the present research is to define clinical risk factors of ischemic liver injury after AAD surgery using single center cohort of patients. In particular, we hope to create and internally validate a model to predict the individual risk of ILI.

## Methods

### Patients and Data Collection

**Figure 1:**
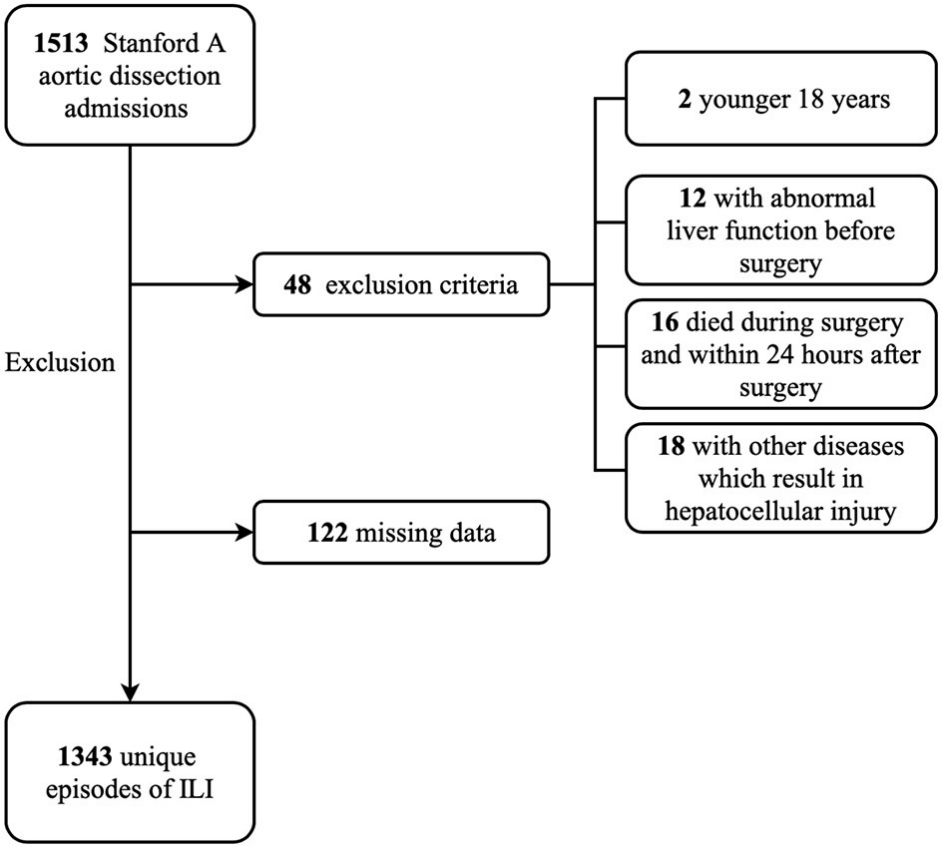
Flow diagram of the exclusion criteria. ILI, ischemic liver injury.

Between January 1, 2015, and October 30, 2020, we retrospectively reviewed 1,513 patients who were diagnosed as Stanford A aortic dissection and underwent aortic surgery in Beijing Anzhen Hospital, Capital Medical University. The exclusion criteria (Figure 1) were (1) patients who were younger 18 years, (2) patients with abnormal liver function before surgery, (3) patients who died during surgery and within 24 h after surgery, (4) patients with other diseases which result in hepatocellular injury, (5) missing data. Finally, 1,343 patients were included in analytic cohort. The study was approved by the Institutional Ethics Committee of the Beijing Anzhen Hospital.

Demographic and clinical data were collected, including age, sex, BMI, medical history, pre-operative alanine aminotransferase (ALT), the peak level of post-operative ALT, pre-operative serum creatinine (Cr), pre-operative red blood cell (RBC) count, coagulation function (i.e., INR, D-Dimer), cardiopulmonary bypass (CPB) time, aortic cross-clamp time (ACT), deep hypothermic circulatory arrest (DHCA) time, blood loss volume, blood transfusion volume, atrial fibrillation within 24 h after operation, ICU stay time, and all-cause mortality in hospital. The primary outcomes of interest were ischemic liver injury after cardiac surgery.

### Statistical Analysis

Categorical variables were reported as whole numbers and proportions, and continuous variables were reported as medians with interquartile ranges (IQRs). Clinical variables associated with the risk factors for ILI were based on clinical importance and predictors identified in previously published articles (2, 21, 22, 24). We use the variance inflation factor (VIF) to evaluate all variables for collinearity. Continuous predictors (i.e., CPB time and Cr) were categorized after being assessed using median or mean value.

We randomly divided 1,343 patients into the training (940 cases) and internal test (403 cases) sets. The significance of each variable was assessed by univariable logistic regression analysis in the training cohort. The variables with *P* < 0.1 were entered into the multivariable logistic regression analysis to identify the risk factors. Next, a nomogram to predict the probability of ILI rates after cardiac surgery was constructed by using the rms package of R, version 4.0.3 (http://www.r-project.org/). The regression coefficients in multivariate logistic regression are proportionally converted to a point scale, and the total points are transformed into predicted probabilities (25).

The performance of the nomogram was evaluated by discrimination and calibration. The discriminative ability of the model was reflected by the area under the receiver operating characteristic curve (is equivalent to the C statistics). Calibration was performed by a visual calibration plot via 1,000 bootstrap samples to decrease the overfit bias (26). An insignificant Hosmer-Lemeshow (HL) test also implies good calibration (*P* > 0.05). In addition, we calculated the misdiagnosis rate by using 10-fold cross-validation. The statistical analysis and graphics were performed with R 4.0.3. All tests were 2-tailed, and *P* < 0.05 was considered to be statistically significant.

## Results

### Demographic and Clinical Characteristics

In two groups of cases, there were 99 cases of simple aortic arch replacement, 23 cases of ascending aorta and aortic arch replacement, 898 cases of total-arch replacement and elephant trunk surgical procedure, and concurrent operations included: 539 cases of Bentall, 4 cases of David, 3 cases of Wheat's, and 59 cases of coronary artery bypass grafting.

The median patient age was 49 years (IQR, 41–56 years), and 23% (216 of 940) of the patients were female. In total, 71% (66 of 93) of the ILI patients were complicated with hypertension, and 30% (28 of 93) of the ILI patients had valvular disease. There were 6% (52 of 940) of the patients had underwent cardiac surgery before. The pre-operative RBC count lower than 3.31T/L was observed in 49% (415 of 847) of non-ILI patients and 63% (59 of 93) of ILI patients. The pre-operative serum creatinine was 120.5 μmol/L (IQR, 87.2–169.1 μmol/L) in ILI patients, while only 93.9 μmol/L (IQR, 73.1–125.8 μmol/L) in Non-ILI patients. Compared to non-ILI patients (*n* = 847), 59% of ILI patients (*n* = 93) more blood transfused (washed red blood cells, WRBC) during operation. ILI patients had significantly higher intraoperative lactic acid level (median of 5.8 vs. 4.3 mmol/L, *P* < 0.001). They were more likely to have atrial fibrillation (26 vs. 12%, *P* < 0.001) within 24 h after surgery. The CPB time exceeded 199 min was observed in 49% (418 of 847) of Non-ILI patients and 62% (58 of 93) of ILI patients. In total, 26% (24 of 93) of the patients underwent surgery with long ACT time (>140 min) in ILI patients and 17% (147 of 847) in Non-ILI patients.

In our study cohort, 93 patients had peak ALT levels exceeding 10 times of the upper limit of normal value and were diagnosed as ILI. The median peak ALT level was 897 U/L (IQR, 629–2,195 U/L) and 104 U/L (IQR, 49–176 U/L) in the ILI and Non-ILI patients, respectively. The all-cause mortality associated with ILI was 28% (26 of 93). In addition, ILI patients had longer ICU stay time (median of 99 vs. 46 h, *P* < 0.01). More characteristics of patients are presented in Table 1.

**Table 1:** Baseline characteristic of the 1,343 AAD patients.

**Baseline variable**	**Total (*n* = 940)**	**Non-ILI (*n* = 847)**	**ILI (*n* = 93)**	***P*-value**
Age (years)	49.0 (41.0, 56.0)	49.0 (41.0, 56.0)	51.0 (42.0, 60.0)	0.257
BMI (Kg/m^2^)	26.0 (23.6, 28.4)	26.0 (23.7, 28.5)	26.0 (23.2, 27.7)	0.290
Gender, female (%)	216 (23)	199 (23)	17 (18)	0.315
**Medical history**				
Cardiac surgery history (%)	52 (6)	50 (6)	2 (2)	0.206
Hypertension (%)	715 (76)	649 (77)	66 (71)	0.278
Hyperlipidemia (%)	55 (6)	50 (6)	5 (5)	1.000
Valvular disease (%)	307 (33)	279 (33)	28 (30)	0.663
**Preoperative test**				
LVEF (%)	62 (58, 66)	62 (58, 66)	60 (59, 66)	0.611
**CT (Debakey type, %)**				0.811
Debakey type I	632 (67)	571 (67)	61 (66)	
Debakey type II	308 (33)	276 (33)	32 (34)	
RBC <3.31 (T/L, %)	474 (50)	415 (49)	59 (63)	0.011
INR	1.2 (1.1, 1.3)	1.2 (1.1, 1.3)	1.3 (1.1, 1.5)	<0.001
ALT (U/L)	19.0 (14.0, 30.0)	19.0 (13.0, 30.0)	19.0 (15.0, 33.0)	0.138
Creatinine (μmol/L)	95.4 (74.2, 129.2)	93.9 (73.1, 125.8)	120.5 (87.2, 169.1)	<0.001
D-Dimer (ng/ml)	1,751.5 (934.3, 2,733.3)	1,723.0 (909.5, 2,660.5)	2,113.0 (1231.0, 3,111.0)	0.006
**Intraoperative variables**				
CPB time > 199 (min)	476 (51)	418 (49)	58 (62)	0.023
DHCA time > 25.6 (min, %)	366 (39)	337 (40)	29 (31)	0.133
ACT > 140 (min)	171 (18)	147 (17)	24 (26)	0.062
Blood loss > 1,200 (ml)	447 (48)	391 (46)	56 (60)	0.014
RBCS > 2.47 (U)	262 (28)	234 (28)	28 (30)	0.701
Plasma > 213( ml)	344 (37)	303 (36)	41 (44)	0.143
WRBC (%)	468 (50)	413 (49)	55 (59)	0.073
**Lactic acid level (mmol/L)**				<0.001
<3	223 (24)	214 (25)	9 (10)	
≥3 and <4.5	259 (28)	233 (28)	26 (28)	
≥4.5 and <7	241 (26)	218 (26)	23 (25)	
≥7	217 (23)	182 (21)	35 (38)	
AF (%)	125 (13)	101 (12)	24 (26)	<0.001
Death (%)	93 (10)	67 (8)	26 (28)	<0.001
ALT-max (U/L)	119.0 (53.0, 234.5)	104.0 (49.0, 176.0)	897.0 (629.0, 2195.0)	<0.001
ICU detention time (hour)	48.0 (24.0, 115.0)	46.0 (23.0, 107.5)	99.0 (40.0, 201.0)	<0.001

*LVEF, left ventricular ejection fraction; CT, whether the dissection involves the abdominal aorta; RBC, red blood cell; INR, International Normalized Ratio; ALT, alanine aminotransferase; CPB, cardiopulmonary bypass; DHCA, deep hypothermic circulatory; ACT, aortic cross-clamp time; RBCS, the transfusion of red cells suspension; Plasma, the transfusion of plasma; WRBC, the transfusion of washed red blood cells; AF, atrial fibrillation within post-operative 24 h; Death, in-hospital all-cause die; ALT-max, the peak value of post-operative alanine aminotransferase*.

The normal ranges of diagnostic tests: RBC (4.3–5.8 ^*^ 10^12/L^), INR (0.8–1.2), ALT (9–50 U/L), Creatinine (57–111 μmol/L), D-Dimer (0–243 ng/ml), Lactic acid (0.5–1.6 mmol/L).

### Selected Factors for Model

The variables used in this analysis were clinical important characteristics and proved risk factors. The results of univariable logistic regression analysis are listed in Table 2. On multivariable analysis, the variables of pre-operative serum creatinine (OR, 1.01; 95% CI, 1.00–1.01; *P* = 0.021), pre-operative RBC count (OR, 1.96; 95% CI, 1.24–3.13; *P* = 0.004), intraoperative lactic acid level (OR, 1.44; 95% CI, 1.17–1.78; *P* < 0.001), ACT (OR, 1.71; 95% CI, 1.06–2.98; *P* = 0.026), the transfusion of WRBC (OR, 1.62; 95% CI, 1.09–2.70; *P* = 0.021), atrial fibrillation (OR, 2.48; 95% CI, 1.44–4.15; *P* < 0.001) were independently associated with ILI (Table 2).

**Table 2:** Logistic multivariable regression analysis showing the risk variables of ILI after AAD surgery.

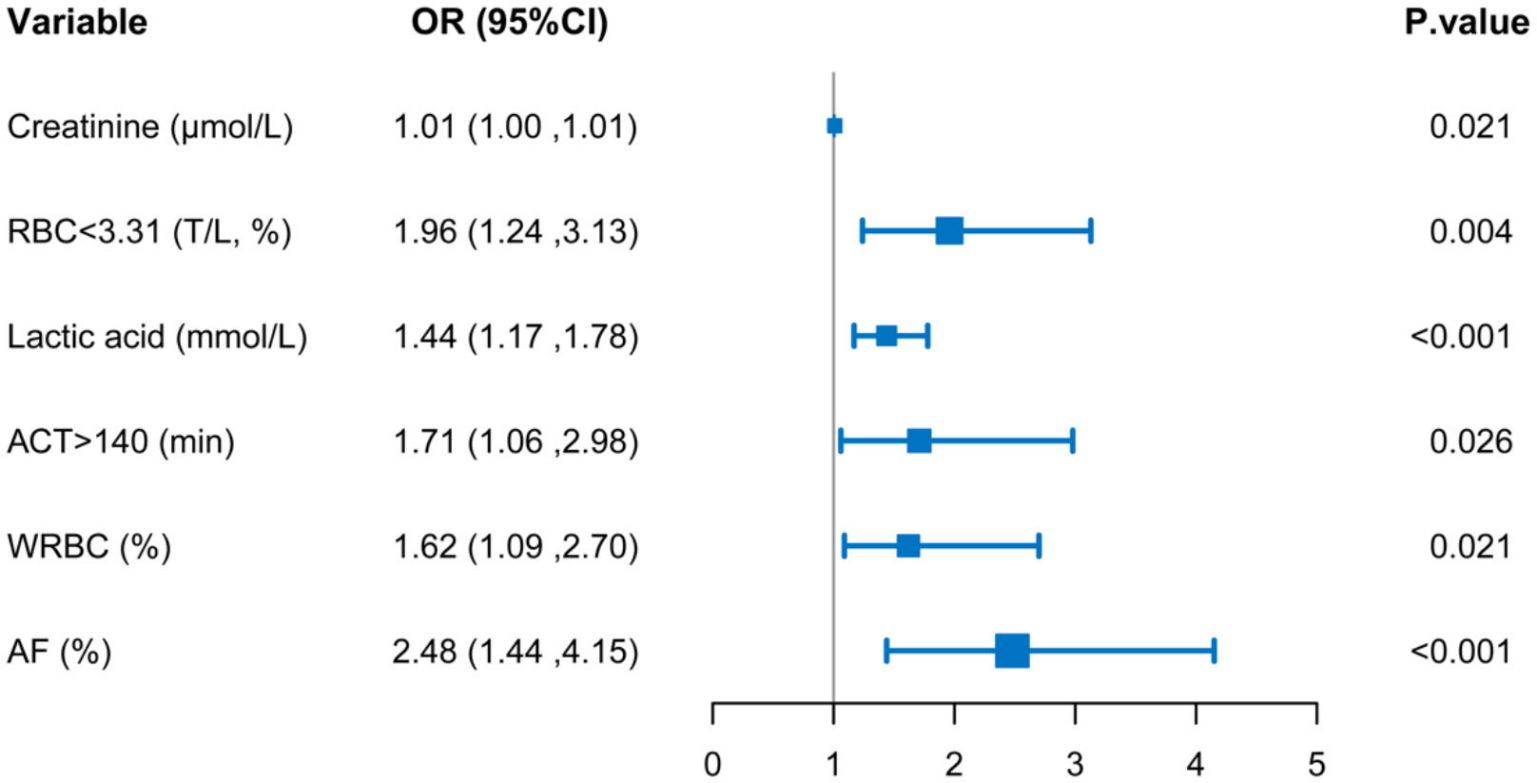

*OR, Odds ratio; CI, confidence interval; RBC, red blood cell; ACT, aortic cross-clamp time; WRBC, the transfusion of washed red blood cells; AF, atrial fibrillation within post-operative 24 h*.

### Nomograms and Model Performance

**Figure 2:**
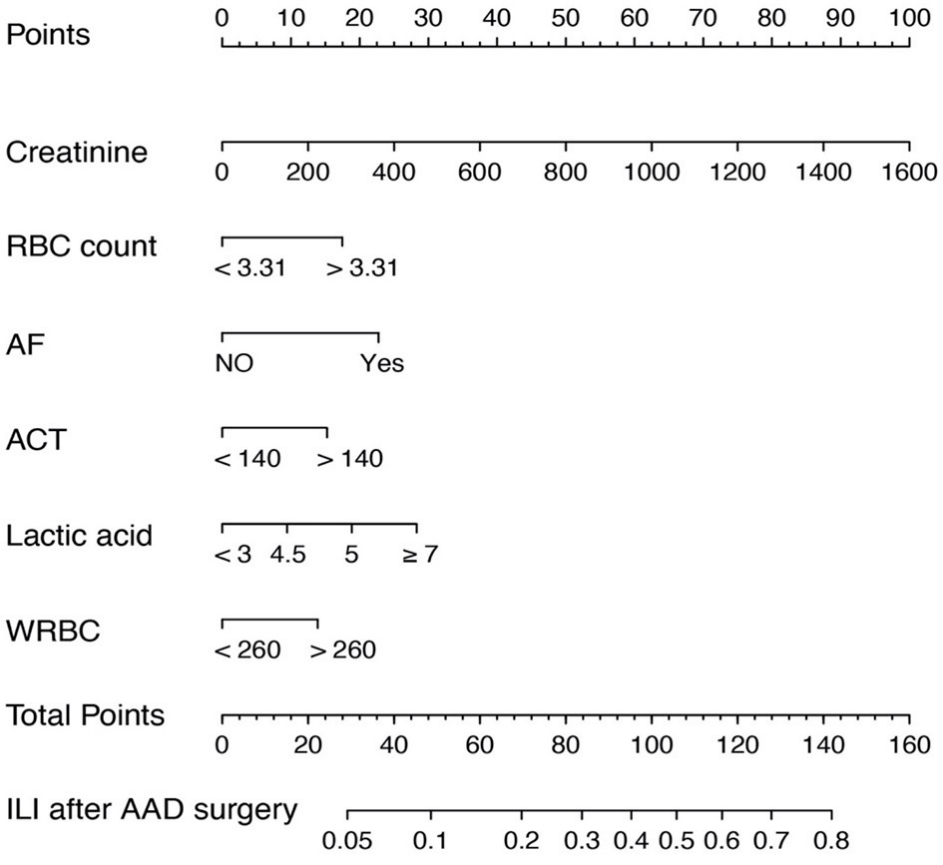
Nomogram predicting ILI risk in patients after AAD surgery. The nomogram to predict the risk of ILI for patients after AAD surgery was created based on 6 independent prognostic factors. The value of each of variable was given a score on the point scale axis. A total score could be calculated by adding each single score, and we can estimate the probability of ILI by projecting the total score to the lower total point scale.

In accordance with the multivariable logistic regression analysis, a nomogram was created to predict ILI after cardiac surgery, including 6 significant risk factors: pre-operative serum creatinine, pre-operative RBC count, intraoperative lactic acid level, ACT, the transfusion of WRBC, atrial fibrillation (Figure 2). A total score reached by summing up the single scores, was used to estimate the probability of ILI.

**Figure 3:**
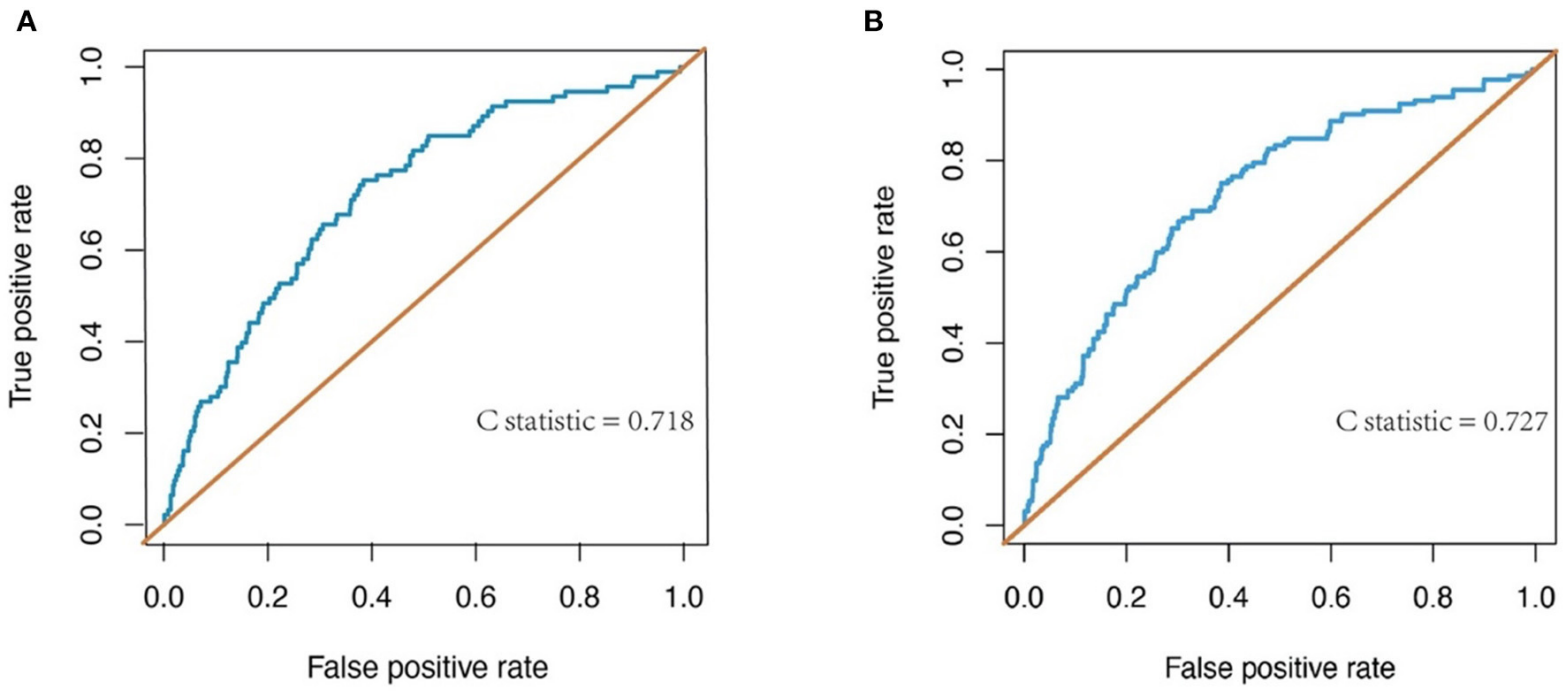
Receiver-operating characteristic (ROC) curve for evaluating the discrimination performance of the model in both the training and validation cohorts. **(A)** ROC curve to evaluate discrimination performance in the training cohort; C-statistic was 0.718. **(B)** ROC curve for evaluating discrimination performance in the validation cohort; C-statistic was 0.727.

**Figure 4:**
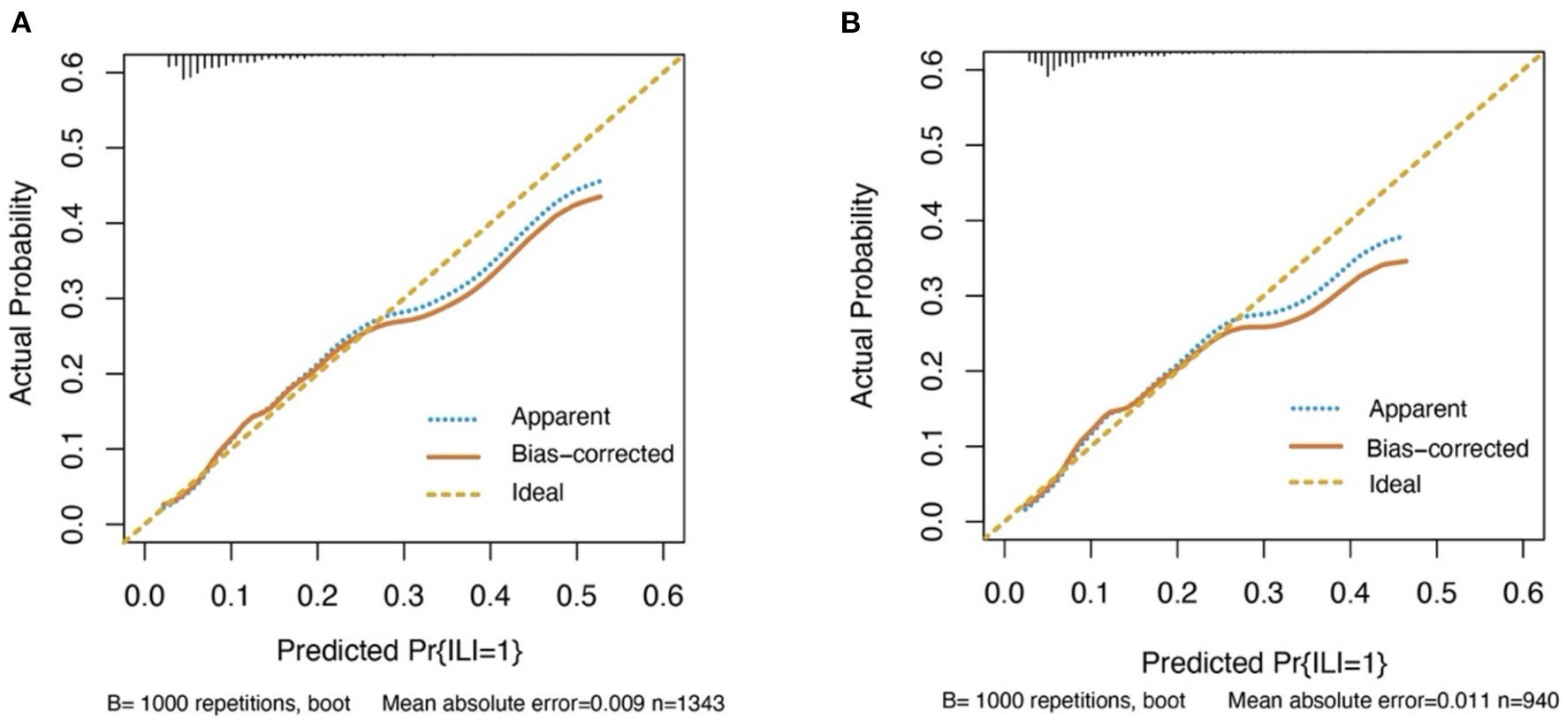
Calibration curves for the prediction model in both cohorts. The curves describe the calibration of the nomogram in terms of agreement between predicted risks (x-axes) and actual outcomes (y-axes). The diagonal line indicates perfect prediction by an ideal model. The curve indicates the performance of the model. **(A)** Training cohort. **(B)** Validation cohort.

The discrimination of the predict model in the training cohort was assessed using an unadjusted C statistic of 0.718 (95% CI, 0.665–0.771) and a bootstrap-corrected C statistic of 0.701. In the validation cohort, the model represented a C statistic of 0.727 (95% CI, 0.640–0.816) for the estimation of ILI risk (Figure 3). The calibration plots showed that the predicted probabilities of ILI fitted well with the actual prevalence rates (Figure 4) and the HL test (*P* = 0.637 in the training cohort, *P* = 0.872 in the validation cohort) also demonstrated the good calibration. In addition, 10-fold cross-validation in full data set of the predict model demonstrated that the average misdiagnosis rate was 9.79% and the lowest misdiagnosis rate was 9.56%.

## Discussion

Previous studies have demonstrated multiple, but poorly studied risk factors for ILI, after Stanford A aortic dissection (AAD) surgery. In the present study, the formal nomogram, which demonstrated that pre-operative serum creatinine, pre-operative RBC count <3.31 T/L, aortic cross-clamp time >140 min, intraoperative lactic acid level, the transfusion of WRBC, atrial fibrillation within post-operative 24 h might increase the risk of ILI after AAD surgery. In our nomogram, the greater contributors to the risk of ILI were the intraoperative lactic acid level and atrial fibrillation.

Due to the complexity of surgery, long operation time, extracorporeal circulation (i.e., CPB), and macro trauma, post-operative ILI has higher morbidity and mortality than that reported in other types of ischemic liver injury (23, 27, 28). In this study, we found that the incidence of ILI after AAD surgery was 9.9% and the all-cause mortality was 30.8%. Additionally, researchers (6, 7, 11, 29) have shown that the severity and duration of ischemia as the primary determinants of the prognostic of ILI, and liver damage can influence the outcome of AAD surgery. Therefore, the current study is very useful clinically because the model can predict the post-operative ILI as early as possible. Among the currently available prediction tools, a nomogram is easy to quantify the risk of ILI and has good discrimination and calibration in predicting outcomes (30). As far as we know, no study before has reported the model as we did to assess the risk variables independently for their inclusion in ultimate nomogram for post-operative ILI.

Some researchers (22, 23, 31, 32) have reported that a correlation between female gender, hypertension, diabetes, lower CPB temperature, valvular disease, and ischemic liver injury. In the risk prediction model, we noted no association of these factors with ILI after AAD surgery (Table 1). In contrast, longer aortic cross-clamp time (28, 33), blood transfusion (22, 34) (the transfusion of WRBC), pre-operative serum creatinine (23, 27, 29) have been reported to increase the risk of ischemic liver injury. Indeed, our study suggested that these 3 variables were also significantly associated with ILI after AAD surgery. In addition, we demonstrated that a low pre-operative RBC count (<3.31 T/L), high intraoperative lactic acid level and atrial fibrillation were associated with an increased probability of ILI after AAD surgery.

In the current study, we found the intraoperative lactic acid level is significantly associated with post-operative ILI. Deeb et al. (13) reported that aortic dissection can result in the vital branch arterial stricture, especially the combination of celiac and mesenteric arterial stenosis (35), which may reduce the blood supply to the liver. If not treated timely, liver dysfunction and even infarction may happen caused by ischemia. Moreover, Muraki et al. (36) have reported that the serum lactate level can be a sensitive marker of the mesentery ischemia, and consequently a rapid increase in lactate level can reflect the ischemic liver injury. These studies support our clinical opinion that high intraoperative lactic acid level is an important risk factor for the ILI after AAD surgery.

Dysrhythmias occur frequently in the post-operative period of cardiac surgery, particularly atrial fibrillation, which occurs in 10 to 65% of patients requiring cardiopulmonary bypass (22, 37). During acute atrial fibrillation with rapid ventricular response, the rapid heart rate impairs the diastolic filling time and the effective atrial contraction (22, 38, 39). Additionally, Anter et al. have reported that the onset of atrial fibrillation was significantly associated with worsening of the cardiac index and the New York Heart Association (NYHA) functional class (38). As a result, the cardiac output may reduce 15 ~ 25% (37), which decreases the hepatic blood flow and contribute to the development of ischemic liver injury.

Hepatic congestion and ischemia are common causes of ischemic liver injury. Lee et al. (40) have reported that tissue oedema in various diseases may induce hypoxia. Meanwhile, some researchers (40, 41) suggest that red blood cells have adaptive mechanisms by export of nitrate oxide bioactivity, which can support basic cellular activities in response to hypoxia. In addition, red blood cells play an important role in systemic oxygen transport and can sense the relationship between tissue oxygen demand and oxygen supply (41). Therefore, we propose that pre-operative low red blood cells count may be a risk factor of ILI after AAD surgery. Future researchers can prove this opinion by prospective studies.

The present study has several limitations. Firstly, the samples of our study were from a single institution, the proposed nomogram needs externally validation in future studies. Secondly, we constructed the prediction model retrospectively, a prospective study is required to verify the reliability of this model. Finally, the accuracy of the nomogram has not reached high reliability. If critical clinical decisions are required, there is still a misdiagnosis rate.

## Conclusions

In summary, we have developed and internally validated a nomogram for predicting the risk of ischemic liver injury. The nomogram provides individual predictions of each patient, which can help improving treatment suggestions for patients with ILI after AAD surgery.

## Data Availability Statement

The raw data supporting the conclusions of this article will be made available by the authors, without undue reservation.

## Ethics Statement

The studies involving human participants were reviewed and approved by the Ethics Committee of Beijing Anzhen Hospital, Capital Medical University. The Ethics Committee waived the requirement of written informed consent for participation.

## Author Contributions

ML collected, analyzed the data, and wrote the manuscript. WT, WY, TW, and NL reviewed and edited it. All authors contributed to the submitted version.

## Conflict of Interest

The authors declare that the research was conducted in the absence of any commercial or financial relationships that could be construed as a potential conflict of interest.

## Publisher's Note

All claims expressed in this article are solely those of the authors and do not necessarily represent those of their affiliated organizations, or those of the publisher, the editors and the reviewers. Any product that may be evaluated in this article, or claim that may be made by its manufacturer, is not guaranteed or endorsed by the publisher.
